# Supplementary material for: Directional spontaneous emission in photonic crystal slabs
Source: Nanophotonics. 2024 Feb 26;13(11):1963–73. doi: 10.1515/nanoph-2023-0843 (PMC11501751; doi:10.1515/nanoph-2023-0843)
Supplement: Supplementary file 1 — Supplementary Material Details [file j_nanoph-2023-0843_suppl_001.zip › SM/SupplemntaryMaterial-Description.docx]

**Video 1.**

Electric field intensity and S_1_ parameter produce by a linear polarized dipole at the center of the unit cell. The black arrow show the polarization of the dipole at center of the unit cell, which is changing in the video.

**Video 2.**

Electric field intensity and S_1_ parameter produce by a linear polarized dipole at the position **r**= (0, 0.35a). The black arrow show the polarization of the dipole, which is changing in the video.

**Video 3.**

Electric field intensity and S_3_ parameter produce by a circularly polarized dipole at the position **r**= (0, 0.35a). The black circle with arrows show the circular polarization of the dipole, which is changing in the video.

**Video 4.**

Electric field intensity and S_3_ parameter produce by a circularly polarized dipole at the position **r**= (0, a/√3) on the center of smallest air holes. The black circle with arrows show the circular polarization of the dipole, which is changing in the video.
